# Hemodynamic Modeling of Biological Aortic Valve Replacement Using Preoperative Data Only

**DOI:** 10.3389/fcvm.2020.593709

**Published:** 2021-02-09

**Authors:** Florian Hellmeier, Jan Brüning, Simon Sündermann, Lina Jarmatz, Marie Schafstedde, Leonid Goubergrits, Titus Kühne, Sarah Nordmeyer

**Affiliations:** ^1^Charité - Universitätsmedizin Berlin, Institute for Imaging Science and Computational Modelling in Cardiovascular Medicine, Berlin, Germany; ^2^Charité - Universitätsmedizin Berlin, Department of Cardiovascular Surgery, Berlin, Germany; ^3^German Heart Center Berlin, Department of Cardiothoracic and Vascular Surgery, Berlin, Germany; ^4^DZHK (German Center for Cardiovascular Research), Partner Site Berlin, Berlin, Germany; ^5^Berlin Institute of Health (BIH), Berlin, Germany; ^6^German Heart Center Berlin, Department of Congenital Heart Disease, Berlin, Germany; ^7^Einstein Center Digital Future, Berlin, Germany

**Keywords:** aortic valve replacement, virtual intervention, CFD, 4D flow MRI, hemodynamics

## Abstract

**Objectives:** Prediction of aortic hemodynamics after aortic valve replacement (AVR) could help optimize treatment planning and improve outcomes. This study aims to demonstrate an approach to predict postoperative maximum velocity, maximum pressure gradient, secondary flow degree (SFD), and normalized flow displacement (NFD) in patients receiving biological AVR.

**Methods:** Virtual AVR was performed for 10 patients, who received actual AVR with a biological prosthesis. The virtual AVRs used only preoperative anatomical and 4D flow MRI data. Subsequently, computational fluid dynamics (CFD) simulations were performed and the abovementioned hemodynamic parameters compared between postoperative 4D flow MRI data and CFD results.

**Results:** For maximum velocities and pressure gradients, postoperative 4D flow MRI data and CFD results were strongly correlated (*R*^2^ = 0.75 and *R*^2^ = 0.81) with low root mean square error (0.21 m/s and 3.8 mmHg). SFD and NFD were moderately and weakly correlated at *R*^2^ = 0.44 and *R*^2^ = 0.20, respectively. Flow visualization through streamlines indicates good qualitative agreement between 4D flow MRI data and CFD results in most cases.

**Conclusion:** The approach presented here seems suitable to estimate postoperative maximum velocity and pressure gradient in patients receiving biological AVR, using only preoperative MRI data. The workflow can be performed in a reasonable time frame and offers a method to estimate postoperative valve prosthesis performance and to identify patients at risk of patient-prosthesis mismatch preoperatively. Novel parameters, such as SFD and NFD, appear to be more sensitive, and estimation seems harder. Further workflow optimization and validation of results seems warranted.

## Introduction

Nine thousand eight hundred and twenty nine surgical isolated aortic valve replacements (AVR) and at least 13,279 transcatheter aortic valve implantations (TAVI) were performed in Germany in 2018. Approximately 10% of the surgical AVRs used mechanical prostheses, with the remainder being tissue valve prostheses of animal or human origin, while TAVI currently exclusively use tissue valve prostheses (1). In general, the decision whether to use a surgical or transcatheter approach as well as the choice of prosthesis material is a clinical one and considers, among other factors, age, life expectancy, risk of surgery, indications and contraindications for anticoagulation, other preexisting cardiovascular conditions, and the will of the patient.

A clinically relevant problem after AVR is patient-prosthesis mismatch (PPM), typically classified by indexed effective orifice area (IEOA), which is defined as the effective orifice area (EOA) of the prosthesis in cm^2^ normalized with the body surface area in m^2^. For echocardiography-based IEOA, values above 0.85 cm^2^/m^2^ are commonly classified as no PPM, values between 0.65 cm^2^/m^2^ and 0.85 cm^2^/m^2^ as moderate PPM, and values below 0.65 cm^2^/m^2^ as severe PPM (2). PPM has been shown to lead to increased morbidity and mortality as well as decreased left ventricular mass regression following AVR (2, 3). Mechanistically, PPM leads to increased cardiac workload through higher-than-normal postoperative aortic valve resistance and may require redo AVR. Other parameters to assess the effect of prosthesis size on hemodynamics are maximum velocity and pressure gradient across the aortic valve. Please note that the term “pressure gradient” is used in its medical sense, describing what is essentially a pressure difference. Parameters quantifying aortic hemodynamics include secondary flow degree (SFD) and normalized flow displacement (NFD). SFD quantifies the amount of secondary flow while incorporating vessel orientation, unlike helicity, for example. It has previously been used to characterize aortic flows in the presence of valve prostheses (4), to evaluate hemodynamic reactions to exercise (5), and is related to wall shear stress (6). Regarding NFD, several groups identified an association between increased NFD and aortic dilation (7–9), while others found it to be related to left ventricular remodeling (10). Compared to jet angle, NFD seems to be a more reliable measure of flow eccentricity (11).

Maximum velocity and pressure gradient are typically estimated using echocardiography in clinical settings. While inexpensive and readily available, echocardiography-based determination of velocities and pressure gradients is heavily operator-dependent and only able to capture velocity components in the direction of wave propagation. Current research by Adriaans et al. indicates that time-resolved phase-contrast magnetic resonance imaging (4D flow MRI) offers more consistent measurement of velocity fields (12).

Given that the hemodynamic result of AVR can currently only be assessed retroactively, it would be beneficial to develop computational approaches capable of providing hemodynamic estimates ahead of surgery, which could help in improving therapy planning and outcomes by providing additional information to clinicians. Computational fluid dynamics (CFD) simulations provide a way to predict hemodynamic parameters, and predictive modeling of diseases of the aortic, coronary, and cerebral vasculature using CFD is a field of active research (13–15). Some current CFD models provide diagnostic accuracy comparable to clinical diagnostic procedures, e.g., in the estimation of fractional flow reserve (16, 17). The publications by Morris et al. (13) and Itatani et al. (14) also contain well-written introductions to CFD methods used in cardiovascular medicine, which may be helpful to readers unfamiliar with CFD. The online version of the former publication also contains two videos on the topic. There are various approaches for simulating aortic valve prosthesis hemodynamics, ranging from 2D models to spatially and temporally resolved FSI simulations including prosthesis leaflet dynamics (18, 19). Most studies focus on estimating specific properties, such as thrombogenicity of mechanical bi-leaflet prostheses, or comparing the hemodynamics of different prostheses types (4, 20). Numerical studies on biological prostheses usually focus on transcatheter implantation and specific aspects of this intervention type, e.g., the predictability of paravalvular leakage (21). In contrast, this study tries to provide a method capable of estimating the immediate hemodynamic outcome after AVR in a homogenous cohort.

Given their frequent clinical use and the fact that they exhibit a natural orientation during implantation, we decided to focus on surgically implanted, biological aortic valve prostheses. This study aims to provide a method capable of estimating clinically relevant hemodynamic aortic valve parameters in patients receiving surgical AVR with a biological valve prosthesis, using only information available preoperatively. This was done by using preoperative MRI data of the patients to reconstruct the geometry of the aorta and perform virtual AVR. Subsequently, CFD simulations were performed on the virtual AVR geometries and the relevant hemodynamic parameters were computed from the CFD results. The CFD-based hemodynamic parameters were then compared with the same parameters computed from postoperative 4D flow MRI data. We decided to compare CFD against 4D flow MRI instead of echocardiography due to the increased reproducibility and consistency of 4D flow MRI mentioned above.

## Patients and Methods

The data used in this study was acquired during the SMART study (ClinicalTrials.gov identifier NCT03172338, German Heart Center Berlin, ethics committee approval ID EA2/133/14, 10.12.2015). Written informed consent was obtained from all patients.

### Magnetic Resonance Imaging Acquisition

We used data from 10 patients, who underwent surgical AVR at the German Heart Center Berlin. Patient characteristics are summarized in Table 1. All patients received a Carpentier-Edwards Perimount Magna Ease aortic valve prosthesis (Edwards Lifesciences, Irvine, USA). Pre- and post-operative anatomical MRI imaging was performed on a 1.5 T Achieva MRI scanner (Philips Medical Systems, Hamburg, Germany) with a typical reconstructed voxel size of 0.80 × 0.80 × 2.00 mm. Anatomical imaging data was obtained during diastole. 4D flow MRI data was reconstructed at a typical voxel size of 2.80 × 2.25 × 2.25 mm with a temporal resolution of 25 phases per cardiac cycle.

**Table 1:** Patient characteristics.

**Characteristic**	**Median (range) or ratio**
Age [years]	64 (57–72)
Sex [female]	3/10
Weight [kg]	95 (70–110)
Height [cm]	182 (164–198)
Body surface area [m^2^]	2.19 (1.80–2.42)
Aortic valve stenosis, preoperative	10/10
Aortic valve insufficiency, preoperative	4/10 (all classified as mild)

**Figure 1:**
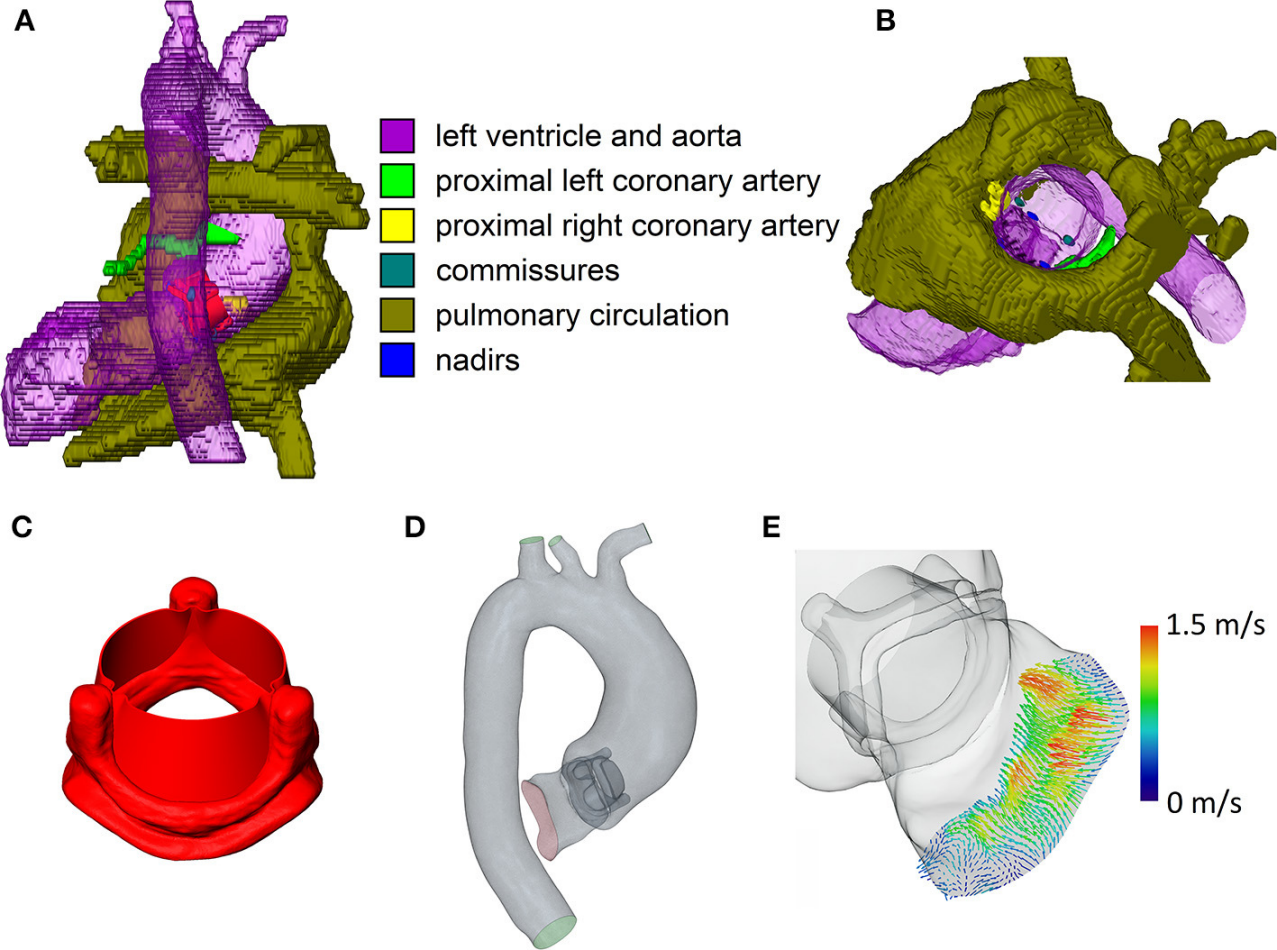
Relevant steps of the virtual intervention process. **(A)** Example of virtual operating field, dorsal view. **(B)** Example of the virtual operating field, oblique view, ascending aorta and aortic arch removed. **(C)** 3D model of the open 23 mm valve prosthesis. **(D)** Example of the final CFD domain used for simulation. **(E)** 4D flow MRI inlet profile for CFD simulations. Note that the images are not from the same patient.

Based on the anatomical imaging data, the left ventricular outflow tract (LVOT), the thoracic aorta, and the most proximal parts of the brachiocephalic, left common carotid, and left subclavian artery were manually segmented using ZIBAmira (version 2015.28, Zuse Institute Berlin, Berlin, Germany). Additionally, the nadirs and commissures of the native aortic valve, the ostia and most proximal parts of the coronary arteries, the anterior leaflet of the mitral valve, and the native aortic valve were segmented approximatively to aid orientation during virtual intervention (Figures 1A,B).

### Computational Fluid Dynamics

Geometries of the Perimount Magna Ease prosthesis and its opened leaflets were kindly provided to us by Claudio Capelli (Institute of Cardiovascular Science, University College London, London, UK). A model of the opened prosthesis was then constructed from the prosthesis frame and leaflet models (Figure 1C); the resulting model of the open prosthesis was subsequently scaled to approximate the different sizes offered by the manufacturer (19–29 mm in 2 mm intervals). Virtual interventions consisted of positioning a 3D model of an opened Perimount Magna Ease prosthesis in the preoperative vessel geometry, subsequent intersection of the prosthesis and vessel geometries, and cleanup of the resulting geometry using STAR-CCM+ (version 13.02, Siemens Digital Industries Software, Plano, USA) and MeshLab (version 2016.12, Visual Computing Lab, Istituto di Scienza e Tecnologie dell'Informazione, Pisa, Italy) to produce a manifold and topologically correct surface geometry (Figure 1D). Choice of prosthesis size and placement was performed by a cardiac surgeon with experience in surgical AVR. In cases where the prosthesis size virtually implanted by the surgeon was different from the one used during the actual surgery, a second geometry was created using the prosthesis size used during surgery, placed in the same position as the first prosthesis. Table 2 shows the prosthesis sizes for all patients.

**Table 2:** Prosthesis size, peak systolic flow rate, and Reynolds number for all patients.

**Patient**	**Nominal prosthesis size, virtual intervention [mm]**	**Nominal prosthesis size, actual surgery [mm]**	**Peak systolic inlet flow rate [ml/s]**	**Reynolds number at the prosthesis, actual size [–]**
I	23	23	354	6,997
II	29	27	430	7,240
III	25	25	394	7,164
IV	25	23	539	10,653
V	25	23	367	7,253
VI	25	23	235	4,645
VII	27	23	407	8,044
VIII	25	21	296	6,407
IX	25	25	412	7,491
X	23	25	464	8,437

The resulting geometry was then used to perform computational fluid dynamics (CFD) simulations using STAR-CCM+. Peak systolic, stationary boundary conditions were set using 4D flow MRI profiles for the LVOT inlet (Figure 1E) and 4D flow MRI flow rates for the descending aorta outlet. Table 2 shows inlet flow rates and Reynolds numbers for all patients. Reynolds numbers were calculated at the narrowest part of the prostheses using the rheological model's infinite shear viscosity μ_∞_ = 0.0035 Pa·s. Flow profiles were extracted from the 4D flow MRI data with MEVISFlow (version 11.0, Fraunhofer MEVIS, Bremen, Germany). The remainder of the flow was distributed among the branching vessels of the aortic arch using Murray's law and requiring that the brachiocephalic artery receives the same flow rate as the left common carotid and left subclavian artery together. Inlet turbulence intensity was set at 5%, similar to values measured by Isaaz et al. *in vivo* (22). An implicit finite volume solver using a shear stress transport k-ω turbulence model was used to solve the Navier–Stokes and continuity equations, which describe the flow field. Blood was modeled as a constant density (1,050 kg/m3) Carreau–Yasuda fluid (zero shear viscosity μ_0_ = 0.16 Pa·s, infinite shear viscosity μ_∞_ = 0.0035 Pa·s, power constant *n* = 0.2128, transition parameter *a* = 0.64, relaxation parameter λ = 8.2 s); for a discussion of viscosity models in aortic CFD, refer to Karimi et al. (23). The boundaries of the flow domain, i.e., the aortic wall and the valve prosthesis, were modeled as rigid. Aortic geometries were modeled in their diastolic state. Based on a convergence study, the base mesh size was chosen at 0.5 mm, at and below which mesh resolution did not relevantly influence the evaluated hemodynamic parameters. A blended wall function approach was used for wall treatment. Cell counts varied from 2.85 to 5.09 million per geometry, depending on the physical size of the computational domain. Simulations were performed on a Xeon E5-2630 v4 CPU (Intel, Santa Clara, USA); the modeling workflow took approximately a week per patient, including segmentation, geometry preparation, virtual intervention, and simulation.

### Hemodynamic Parameters

Evaluated hemodynamic parameters were v_max_ (maximum flow velocity in the valve jet), dp_max_ (maximum pressure gradient across the aortic valve), SFD (secondary flow degree), and NFD (normalized flow displacement). dp_max_ of 4D flow MRI and CFD data was estimated from v_max_ using the commonly used, strongly simplified version of the Bernoulli equation dp_max_ = 4·v_max_^2^ with the units of v_max_ being m/s and the unit of dp_max_ being mmHg (24). Note that while this estimate and its derivatives are commonly and successfully used in clinical decision making and guidelines, they do not accurately reflect the actual physical pressure drop across the aortic valve. For a discussion of pressure recovery in aortic stenoses and prostheses, refer, for example, to Baumgartner et al. (25) or Dohmen et al. (26). SFD and NFD are scalar parameters calculated from three vessel cross sections, which were placed at the sinotubular junction, directly proximal of the brachiocephalic artery and in the middle between the two previous sections (i.e., in the middle of the ascending aorta). SFD is defined as the mean in-plane velocity divided by the mean through-plane velocity. NFD is defined as the flow displacement normalized by the vessel diameter, with the flow displacement being the distance between the vessel center and the center of the flow. We used the hydraulic vessel diameter (4 times cross-sectional area divided by perimeter) for normalization. We also estimated MRI- and CFD-based IEOA for each patient. EOA was estimated using the respective dp_max_ and the EOA equation used by Weese et al. (27). The EOA estimate was then normalized using patient body surface area, to obtain an estimate of IEOA. Additionally, streamlines were calculated to visualize the flows and allow qualitative comparison. Parameters were extracted, calculated, and visualized using MEVISFlow, ZIBAmira and MATLAB (version 2017b, MathWorks, Natick, USA).

Using these hemodynamic parameters, CFD flow fields were compared against postoperative 4D flow MRI data of the patients. To reduce bias, evaluation of v_max_ and placement of the cross sections used to compute SFD and NFD were done by different authors for the CFD and MRI data. The author analyzing CFD data did not have access to the post-interventional MRI data, and the authors analyzing the post-interventional MRI data did not have access to the CFD data. Note that there are 2 CFD data sets for the cases, for which the prosthesis size chosen during the virtual intervention (index _vi_) did not match the one used during actual surgery (index _as_), one for each of the two sizes.

Statistical analysis was performed for v_max_, dp_max_, SFD, NFD, IEOA_mri_, and IEOA_as_, using MATLAB. Since only simple linear regression was performed, the unadjusted coefficient of determination R^2^, which in this case is the square of Pearson's r, was used. Estimates of parameters are indicated using the hat operator, e.g., ${\hat{\text{v}}}_{\text{max}}$. Where specified, the use of median and interquartile range as measures of central tendency and dispersion was based on inspection of histograms of the underlying data.

## Results

In order to identify the hemodynamic results, the following indices are used: _mri_ for the results derived from postoperative 4D flow MRI, _vi_ for the results of the CFD simulations using the prosthesis size determined by the surgeon during virtual intervention, and _as_ for the results of the CFD simulations using the prosthesis size used during the actual surgery performed on the patient. Given the number of patients, we decided to follow a descriptive statistics approach.

### Choice of Prosthesis Size and IEOA

Table 2 summarizes the prosthesis sizes used during virtual interventions and actual surgeries, while Table 3 shows estimated IEOA_as_ for the actual size CFD simulations and IEOA_mri_ for the postoperative 4D flow MRI data. We also calculated the coefficient of determination for linear regression between IEOA_as_ and IEOA_mri_, which was *R*^2^ = 0.52. For three patients, the prosthesis sizes chosen during virtual intervention and actual surgery were identical. The mean absolute error between the prosthesis size chosen during virtual intervention and the prosthesis size actually used was 1.8 mm with the virtual interventions using a larger prosthesis size on average.

**Table 3:** 4D flow MRI- and CFD-based estimated indexed effective orifice areas (IEOA) for all patients.

**Patient**	**Estimated IEOA_mri_ [cm^2^/m^2^]**	**Estimated IEOA_as_ [cm^2^/m^2^]**
I	0.74	1.07
II	0.73	1.14
III	0.59	0.85
IV	0.62	0.80
V	0.91	1.10
VI	0.84	0.96
VII	0.66	0.87
VIII	0.70	0.96
IX	1.00	1.11
X	0.76	0.92

### Pressure Gradients and Maximum Velocities

**Figure 2:**
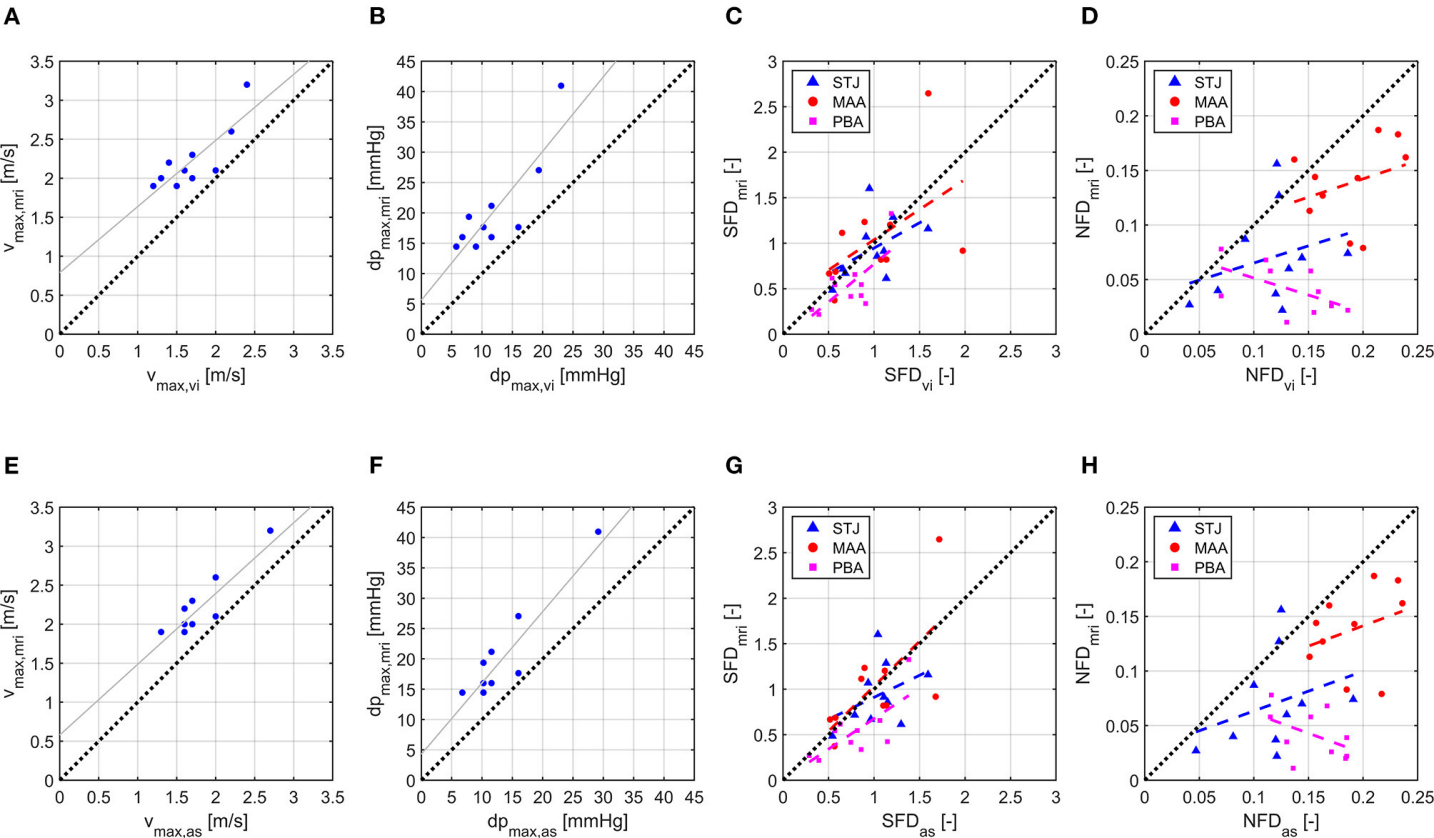
Scatter and linear regression plots for v_max_, dp_max_, SFD, and NFD. **(A)** v_max,vi_ vs. v_max,mri_. **(B)** dp_max,vi_ vs. dp_max,mri_. **(C)** SFD_vi_ vs. SFD_mri_. **(D)** NFD_vi_ vs. NFD_mri_. **(E)** v_max,as_ vs. v_max,mri_. **(F)** dp_max,as_ vs. dp_max,mri_. **(G)** SFD_as_ vs. SFD_mri_. **(H)** NFD_as_ vs. NFD_mri_. Index _mri_ identifies 4D flow MRI results, _vi_ identifies CFD results using virtual intervention prosthesis size, and _as_ identifies CFD results using actual surgery prosthesis size. For SFD and NFD, least-square lines are plotted separately for each cross section with STJ being the cross section at the sinotubular junction, MAA being the cross section in the middle of the ascending aorta, and PBA being the cross section directly proximal to the brachiocephalic artery.

Figure 2 shows scatter and linear regression plots for v_max_ and dp_max_. Coefficient of determination (R^2^), root mean square error (RMSE), and regression equation for v_max,mri_ vs. v_max,vi_ were *R*^2^ = 0.69, *RMSE* = 0.24 m/s, and ${\hat{\text{v}}}_{max,mri}$ = 0.8478·v_max,vi_+0.7887 m/s, while they were *R*^2^ = 0.75, *RMSE* = 0.21 m/s, and ${\hat{\text{v}}}_{\text{max},\text{mri}}$ = 0.9073·v_max,as_+0.5787 m/s for v_max,mri_ vs. v_max,as_. Median values (interquartile range) of v_max_ were 2.1 m/s (0.3 m/s), 1.7 m/s (0.6 m/s), and 1.7 m/s (0.4 m/s) for v_max,mri_, v_max,vi_, and v_max,as_, respectively. Similarly, the statistics were *R*^2^ = 0.73, *RMSE* = 4.5 mmHg, and d${\hat{\text{p}}}_{\text{max},\text{mri}}$ = 1.227·dp_max,vi_+5.601 mmHg for dp_max,mri_ vs. dp_max,vi_, with *R*^2^ = 0.81, *RMSE* = 3.8 mmHg, and d${\hat{\text{p}}}_{\text{max},\text{mri}}$ = 1.175·dp_max,as_+4.281 mmHg for dp_max,mri_ vs. dp _max,as_. Median values (interquartile range) of dp_max_ were 18 mmHg (5 mmHg), 11 mmHg (8 mmHg), and 12 mmHg (6 mmHg) for dp_max,mri_, dp_max,vi_, and dp_max,as_, respectively.

### Quantitative Description of Hemodynamics

Linear regression results for SFD and NFD are shown in Table 4; the coefficients of determination for SFD and NFD were generally lower than for v_max_ and dp_max_. Median values (interquartile range) of SFD over all cross sections were 0.70 (0.57), 0.88 (0.56), and 0.95 (0.51) for SFD_mri_, SFD_vi_, and SFD_as_, respectively. Median values (interquartile range) of NFD over all cross sections were 0.072 (0.090), 0.148 (0.066), and 0.155 (0.062) for NFD_mri_, NFD_vi_, and NFD_as_, respectively. Additionally, Table 4 shows median values and interquartile ranges of SFD and NFD for each cross section individually. Figure 2 contains scatter and linear regression plots for SFD and NFD, color- and marker-coded by cross-section location.

**Table 4:** Median value, interquartile range (IQR), and linear regression for SFD and NFD.

**Parameter**	**Median (IQR) for _mri_**	**Median (IQR) for _vi_**	**Median (IQR) for _as_**	**R^2^_mri_ vs. _vi_**	**R^2^_mri_ vs. _as_**	**RMSE _mri_ vs. _vi_**	**RMSE _mri_ vs. _as_**
SFD all cross sections	0.70 (0.57)	0.88 (0.56)	0.95 (0.51)	0.38	0.44	0.39	0.37
SFD sinotubular junction	0.89 (0.49)	0.99 (0.45)	1.08 (0.21)	0.25	0.16	0.32	0.33
SFD middle of ascending aorta	0.87 (0.52)	0.99 (0.60)	1.00 (0.56)	0.27	0.46	0.56	0.48
SFD proximal to brachiocephalic artery	0.48 (0.28)	0.77 (0.32)	0.78 (0.49)	0.49	0.53	0.24	0.23
NFD all cross sections	0.072 (0.09)	0.148 (0.066)	0.155 (0.062)	0.23	0.20	0.048	0.049
NFD sinotubular junction	0.065 (0.05)	0.122 (0.040)	0.122 (0.030)	0.08	0.10	0.044	0.044
NFD middle of ascending aorta	0.144 (0.049)	0.192 (0.058)	0.189 (0.054)	0.10	0.10	0.038	0.038
NFD proximal to brachiocephalic artery	0.037 (0.036)	0.141 (0.048)	0.160 (0.054)	0.30	0.20	0.020	0.021

*Index _mri_ identifies 4D flow MRI results, _vi_ identifies CFD results using virtual intervention prosthesis size, and _as_ identifies CFD results using actual surgery prosthesis size. Coefficients of determination (R^2^) and root mean square error (RMSE) are for linear regression between MRI- and CFD-based values; all regressions use the CFD-based values as the independent variable*.

### Qualitative Description of Hemodynamics

**Figure 3:**
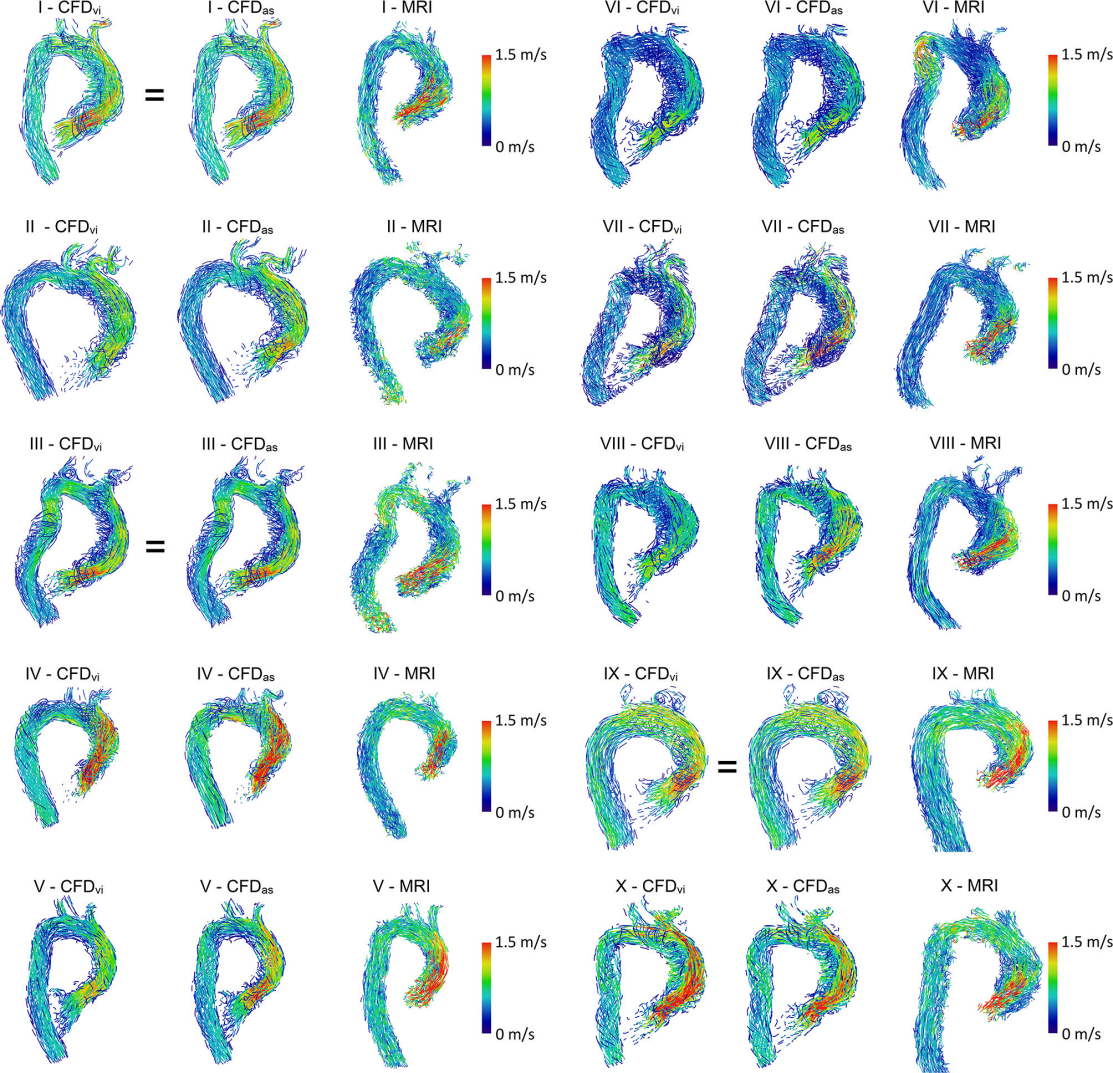
Streamlines of MRI and CFD data. CFD_vi_ denotes simulations using the prosthesis size chosen during virtual intervention, while CFD_as_ denotes simulations using the prosthesis size chosen during actual surgery. Streamlines are color-coded by velocity, right ventrolateral view. Note that the MRI-based streamlines begin at the prosthesis, while the CFD-based streamlines also extend into part of the left ventricle.

For hemodynamic visualization, Figure 3 shows streamlines for all patients. For most patients, the MRI- and CFD-based flow fields agree well, with the main flow features and velocities being comparable. In general, the locations of regions with very low velocities close to stagnation are very similar between both methods. In several patients, these regions are located close to the concave curvature of the ascending aorta (e.g., patients III, VI, VII, and VIII). In patient III, a similar region is identified in the descending aorta by both methods. The orientation of the jet passing through the valve prosthesis, and thus the orientation of the valve prosthesis itself, is also comparable between both methods. Only in patients I and III can a slight deviation between the simulation using the actual size and the MRI measurements be observed.

However, some relevant deviations between measured and calculated hemodynamics can be found. In patient VI, a flow acceleration in the proximal descending aorta is observed in the MRI data. This flow feature is not nearly as pronounced in the CFD data, even though the vessel contours agree well in that region. In the MRI data of patient II, high velocities are observed immediately downstream of the valve prosthesis with a subsequent strong deceleration proximal to the aortic arch. This flow pattern is not found in the CFD data.

The simulations using prosthesis sizes chosen during virtual intervention (CFD_vi_) tend to show lower flow velocities than the simulations using prosthesis sizes chosen during actual surgery (CFD_as_). The visualization also shows that flow patterns are quite similar between CFD_vi_ and CFD_as_.

## Discussion

R^2^ and RMSE indicate good agreement between MRI- and CFD-based values of v_max_ and dp_max_. This suggests that postoperative maximum pressure gradients and velocities can be estimated reasonably well using the current CFD model. The correlation between MRI-based and CFD-based values was generally stronger for the simulations using the actual prosthesis size over the simulations using the prosthesis size chosen during virtual intervention. As the virtual intervention prosthesis sizes were, on average, off by about one size, this observation can most likely be attributed to the mismatch between actual and virtual prosthesis size. Simulations using the prosthesis size chosen during virtual intervention tend to show lower flow velocities, which seems plausible given their, on average, larger orifice areas. The difference in prosthesis size is likely caused by the virtual intervention providing no sizing mechanism similar to the use of a sizing instrument during actual surgeries. This seems to lead to overestimation of the space available for implantation. Furthermore, the suture ring may *in vivo* assume a shape different from the 3D prosthesis model. In practice, the issue of correctly predicting prosthesis size could easily be overcome by being mindful of the tendency to overestimate prosthesis size as well as by performing simulations with multiple possible prosthesis sizes ahead of surgery. This would ensure that the surgeon would preoperatively have CFD results available for all plausible sizes, effectively removing the problem of having to choose the correct prosthesis size.

The underestimation of v_max_ and dp_max_ by CFD might partially be caused by the CFD simulations being based on preoperative MRI profiles. After valve replacement, peak systolic flow likely increases due to reduced valvular resistance, which in turn might explain the relatively higher v_max_ and dp_max_ in the MRI data. Additionally, postoperative prosthesis opening might be subtotal compared to the idealized, fully open prosthesis model used for CFD, resulting in higher v_max_ and dp_max_.

IEOA_mri_ and IEOA_as_ were moderately correlated, indicating that the predicting postoperative IEOA still poses some challenge. While IEOA is a relevant factor, current recommendations also emphasize the importance of flow velocity and pressure gradient in assessing hemodynamic prosthesis performance (28). These parameters exhibit fairly low RMSE of 0.21 m/s and 3.8 mmHg, respectively. By considering all parameters together, one should therefore be able to arrive at a reasonably accurate estimate of postoperative prosthesis performance.

While good agreement between real and virtual intervention was observed for the pressure gradient and the maximum velocity, SFD and NFD featured lower *R*^2^ and worse RMSE. The latter parameters describe the spatially resolved hemodynamics. Even though 4D flow MRI and the velocity information calculated using CFD were registered, to ensure identical orientation of the evaluation cross sections used for both data sets, minor deviations might still be present. Furthermore, SFD and NFD are likely more susceptible to the segmentation of the patient-specific geometry as well as the orientation of the valve prosthesis. Especially the latter parameter will directly affect NFD, as the orientation of the valve prosthesis will directly affect the orientation of its jet-like outflow. As only preoperative information was used for the virtual intervention, the orientation of the virtual and real prosthesis might differ. There might also be some differences between pre- and postoperative anatomies, which could have an influence on SFD/NFD. While we focused on patients receiving isolated AVR, there might still be small anatomical changes associated with surgery and MRI image acquisition, e.g., due to sutures, mobilization of anatomical structures, the prosthesis itself, or subtly different patient posture during postoperative MRI. Another factor possibly influencing SFD and NFD calculation is the use of a RANS-based turbulence model to keep computation time reasonable. RANS-based turbulence models cannot capture all temporal and spatial scales of the flow, which may lead to subtle differences in SFD and NFD. Additionally, the 4D flow MRI resolution was significantly lower than the resolution of the CFD mesh, meaning that some flow scales included in the calculation of CFD-based SFD/NFD might not have been included in the calculation of MRI-based SFD/NFD. Finally, the inlet velocity profiles were measured before AVR and they might subsequently change. It has been shown that the inlet profile can affect the flow distal to the valve prosthesis (6).

Another point regarding NFD is that it has previously been shown to correlate with aortic dilation in patients with high NFD values secondary to strongly eccentric flow, in turn caused by bicuspid aortic valve morphology (7, 8). Burris et al. found an NFD cutoff value of 0.2, above which the aortic growth rate increased significantly in their study (8). Such high values of NFD did rarely occur in our patients, likely due to them receiving the prostheses as well as none of them exhibiting relevant ascending aorta dilation. It is therefore conceivable that the correlation between MRI- and CFD-based NFD would be stronger in patients with higher NFD, in whom constant modeling errors would be relatively smaller.

The MRI- and CFD-based streamlines are quite similar for most patients. In most cases, relevant flow features, such as recirculation zones and the location of prosthesis jet impingement on the aortic wall, were comparable. Velocity magnitudes were mostly similar. The existing small differences could have potentially been caused by segmentation differences, subtly different prosthesis placement, and inertial effects.

The present study demonstrates a method to estimate postoperative hemodynamic parameters after AVR using only preoperative data. Unlike more complex CFD models, which may include unsteady FSI simulations of prosthesis hemodynamics (19, 20), the approach presented in this study uses a less complex model, including rigid walls and stationary boundary conditions. While this means that hemodynamic parameters cannot be evaluated in a time-resolved manner and also neglects dynamic effects, it reduces modeling and computation time and avoids having to estimate unknown elastic properties of the aorta and valve prosthesis. The present method is less well-suited for understanding complex flow phenomena and does not allow analysis of certain features, such as valve prosthesis opening and closing. The workflow can, however, be performed in a clinically reasonable time frame and offers good robustness, which is important for clinical applicability and direct comparison against postoperative 4D flow MRI data. Novel aspects of the present study include the model being solely based on preoperative data, its results being directly compared to postoperative 4D flow MRI data and the virtual intervention being performed by a cardiac surgeon with experience in AVR.

### Limitations

The model used in this study uses a quasi-steady approach to predict the peak systolic hemodynamics after AVR. The aorta and the leaflets of the aortic valve prosthesis were modeled as rigid. Additionally, to evaluate the predictive capabilities of this model, only patient data acquired before AVR was used. These limitations might partially explain the weak-to-moderate correlations of SFD and NFD. For example, replacement of the diseased valve might affect myocardial function and thus the LVOT flow profile as well as peak systolic flow rates. Additionally, the surgical implantation will result in small changes to the anatomy of the ascending aorta. There are currently no models to predict either the change in ventricular hemodynamics or the postoperative anatomy. However, the changes in anatomy seem to be limited, as the vessel contours of the ascending aorta in agree well in all cases.

While simulation of the dynamic behavior of aortic valve prostheses is technically feasible, such simulations not only are associated with higher computational costs but also require estimation of patient-specific material properties. Furthermore, either the transient LVOT inflow profile must be measured, or the left ventricular contraction must be modeled. All these steps introduce additional uncertainty due to model assumptions and additional measurements.

## Conclusion

The results demonstrate that, using the approach presented in this study, MRI-based maximum pressure gradients and velocities across biological aortic valve prostheses can be estimated reasonably well from CFD simulations based only on preoperative MRI and clinical patient data. The approach could potentially be used to identify which prosthesis sizes are likely to put individual patients at risk of increased postoperative pressure gradient ahead of surgery and to estimate postoperative prosthesis performance. The workflow is comparatively inexpensive computationally and can be performed within a clinically reasonable time frame. Regarding novel and sensitive hemodynamic parameters like SFD or NFD, further work needs to be undertaken to accurately estimate them in the patient collective described here.

## Data Availability Statement

The raw data supporting the conclusions of this article will be made available by the authors, without undue reservation.

## Ethics Statement

The studies involving human participants were reviewed and approved by Ethikkommission, Charité - Universitätsmedizin Berlin. The patients/participants provided their written informed consent to participate in this study.

## Author Contributions

All roles according to CRediT (contributor roles taxonomy). FH, SN, LG, and TK: conceptualization. FH, JB, LG, MS, and SN: formal analysis. TK, LG, and SN: funding acquisition. SN, SS, FH, LJ, and JB: investigation. FH, SN, JB, and SS: methodology. LG, TK, and SN: supervision. FH and MS: visualization. FH, LG, JB, and SN: writing—original draft. SS, LJ, MS, and TK: writing—review and editing. All authors contributed to the article and approved the submitted version.

## Conflict of Interest

The authors declare that the research was conducted in the absence of any commercial or financial relationships that could be construed as a potential conflict of interest.
